# Communication strategies to promote the uptake of childhood vaccination in Nigeria: a systematic map

**DOI:** 10.3402/gha.v9.30337

**Published:** 2016-02-12

**Authors:** Afiong Oku, Angela Oyo-Ita, Claire Glenton, Atle Fretheim, Heather Ames, Artur Muloliwa, Jessica Kaufman, Sophie Hill, Julie Cliff, Yuri Cartier, Xavier Bosch-Capblanch, Gabriel Rada, Simon Lewin

**Affiliations:** 1 Community Medicine Department, University of Calabar, Calabar, Nigeria; 2 Global Health Unit, Norwegian Knowledge Centre for the Health Services, Oslo, Norway; 3 Institute of Health and Society, University of Oslo, Oslo, Norway; 4 Departamento de Saúde, Direcção Provincial de Saúde de Nampula, Nampula, Mozambique; 5 Department of Human Biosciences, Centre for Health Communication and Participation, College of Science, Health and Engineering La Trobe University, Melbourne, Australia; 6 Faculdade de Medicina, Universidade Eduardo Mondlane, Maputo, Mozambique; 7 International Union for Health Promotion and Education, Cedex, France; 8 Swiss Tropical and Public Health Institute, Basel, Switzerland; 9 University of Basel, Basel, Switzerland; 10 Evidence-based Healthcare Program, Pontificia Universidad Católica de Chile, Santiago, Chile; 11 Health Systems Research Unit, South African Medical Research Council, Tygerberg, South Africa

**Keywords:** communication interventions, childhood vaccination, Nigeria

## Abstract

**Background:**

Effective communication is a critical component in ensuring that children are fully vaccinated. Although numerous communication interventions have been proposed and implemented in various parts of Nigeria, the range of communication strategies used has not yet been mapped systematically. This study forms part of the ‘Communicate to vaccinate’ (COMMVAC) project, an initiative aimed at building research evidence for improving communication with parents and communities about childhood vaccinations in low- and middle-income countries.

**Objective:**

This study aims to: 1) identify the communication strategies used in two states in Nigeria; 2) map these strategies against the existing COMMVAC taxonomy, a global taxonomy of vaccination communication interventions; 3) create a specific Nigerian country map of interventions organised by purpose and target; and 4) analyse gaps between the COMMVAC taxonomy and the Nigerian map.

**Design:**

We conducted the study in two Nigerian states: Bauchi State in Northern Nigeria and Cross River State in Southern Nigeria. We identified vaccination communication interventions through interviews carried out among purposively selected stakeholders in the health services and relevant agencies involved in vaccination information delivery; through observations and through relevant documents. We used the COMMVAC taxonomy to organise the interventions we identified based on the intended purpose of the communication and the group to which the intervention was targeted.

**Results:**

The Nigerian map revealed that most of the communication strategies identified aimed to *inform and educate* and *remind or recall*. Few aimed to *teach skills, enhance community ownership, and enable communication*. We did not identify any intervention that aimed to *provide support* or *facilitate decision-making*. Many interventions had more than one purpose. The main targets for most interventions were caregivers and community members, with few interventions directed at health workers. Most interventions identified were used in the context of campaigns rather than routine immunisation programmes.

**Conclusions:**

The identification and development of the Nigerian vaccination communication interventions map could assist programme managers to identify gaps in vaccination communication. The map may be a useful tool as part of efforts to address vaccine hesitancy and improve vaccination coverage in Nigeria and similar settings.

## Introduction

Vaccination has been described as one of the greatest public health achievements of the twentieth century and is widely seen as a worthwhile and cost-effective public health measure (1). More than 3 million child deaths worldwide are said to be prevented each year through vaccination (2, 3). Despite these huge benefits, childhood vaccination programmes face numerous challenges including low and stagnant coverage levels, underutilisation of vaccine services, inadequate sustainable financing, and misleading information on vaccination and its effects (4, 5).

Nigeria has one of the highest rates of under-5 mortality in the world and vaccine-preventable diseases account for approximately 22% of child deaths in the country (6). Though vaccination rates have increased in Nigeria in the last decade, only 52% of eligible children in Southern Nigeria were fully vaccinated in 2013 (7), and in the North, even fewer children (10–27%) were fully vaccinated. These low rates have been attributed partly to vaccine hesitancy, a behaviour influenced by a number of factors, including a lack of trust in the vaccine or the provider, people not perceiving a need for or not valuing the vaccine, poor access, lack of knowledge, rumours, religious beliefs, illiteracy, and other social and political factors (5, 8). Effective communication strategies can address some of these issues by making more people aware of the benefits of immunisation; correcting false beliefs, rumours, or concerns that prevent people from getting immunised; and informing people where and when to get immunised, thereby potentially increasing vaccination rates (9–12).

We define a communication intervention as a purposeful, structured, repeatable, and adaptable strategy to inform and influence community decisions to personal and public health participation, disease prevention and promotion, policy making, service improvement, and research (11, 13). Communication interventions are believed to have contributed to the successes recorded in the polio eradication initiative in Nigeria (14). The country's National Social Mobilisation Working Group, headed by UNICEF, is responsible for developing and coordinating communication strategies for all childhood vaccinations. At state and local levels, social mobilisation committees coordinate these activities which tend to focus on the following three objectives: advocacy, social mobilisation and behavioural change communication (15).

Currently, a wide range of communication interventions are being used in Nigeria. Most of the interventions used are developed at the national level and then implemented locally (16, 17) which may result in inadequate community involvement in their planning and implementation (18). To improve communication, it is important to identify what interventions are being used, where, and for which purposes (19, 20); which communication interventions are effective (12, 19, 21); and how people want to be communicated with (22). To better understand some of these issues, we have developed a global taxonomy of communication interventions. This taxonomy aims to map the communication strategies that are used in a way that identifies the key purposes of each strategy, thereby helping to ensure that these strategies address the most relevant determinants of vaccine hesitancy (20).

This study of childhood vaccination communication in Nigeria forms part of the ‘Communicate to vaccinate 2’ (COMMVAC) project – an international project exploring how to integrate evidence-based communication strategies that are adapted for local conditions into vaccination programmes in selected low- and middle-income countries.

## Study objectives

1. To identify communication interventions in use in Nigeria for childhood vaccination.
2. To map these interventions against the existing COMMVAC taxonomy of interventions to create a specific Nigerian map of interventions organised by purpose and target.
3. To analyse gaps between the COMMVAC taxonomy and the Nigerian map to identify potential communication interventions not presently in use that may address particular issues or purposes.
4. To assess the COMMVAC taxonomy as a research tool for data collection and analysis in the field of vaccination communication.

## Methods

### Study setting

The study was conducted in Nigeria, the most populous country in Africa, with an estimated population of more than 170 million people in 2013****. Administratively, Nigeria is divided into 36 states and the Federal Capital Territory, Abuja. Each state is subdivided into local government areas (LGAs), and each LGA is divided into wards. The people of Nigeria are multi-ethnic, multicultural, and multi-religious.

In Nigeria, the National Primary Health Care Development Agency is charged with the responsibility of effectively controlling vaccine-preventable diseases through the provision of vaccines and immunisation guidelines. The national routine immunisation schedule recommends that all childhood vaccinations are completed by 9 months of age. Apart from the routine immunisation schedule, several rounds of supplemental or mass campaigns are held across the country each year in an effort to eradicate poliomyelitis and other vaccine-preventable diseases. The National Social Mobilisation Working Group and the social mobilisation committees at the state and local government levels are responsible for developing and implementing communication interventions for both routine and campaign activities.

### Study sites

We carried out the study in both rural and urban settings in two states: Cross River in Southern Nigeria, and Bauchi in Northern Nigeria. We selected these two states based on variations in vaccination coverage rates, with rates being lower in Bauchi than in Cross River (with Diphtheria, Pertusis, Tetanus third dose (DPT3)) coverage rates of 12.5 and 76.1% respectively) (7); and variations in terms of vaccine hesitancy, with vaccine refusal rates being much higher in Bauchi, linked to religious and cultural beliefs (23). In addition, Bauchi is one of the 12 polio prevalent states of Northern Nigeria and has been the focus of global and national efforts to eliminate polio and improve vaccination uptake. Considerable resources are being spent there on vaccination communication activities. In contrast, Cross River has remained polio free for the last decade.

Vaccination services are delivered through a wide array of strategies, including routine immunisation and campaigns. Routine immunisation is the foundation through which countries provide access to vaccines and control and eradicate vaccine-preventable diseases. It is a continuous service usually conducted by health workers at fixed posts (healthcare centres), outreach locations, or at mobile clinics on fixed days. In contrast, vaccination campaigns are usually intermittent and involve organised mobilisation of a large number of the population to tackle vaccine-preventable diseases intensively (24–26).

### Study design

This was a qualitative study, using semi-structured interviews, observations, and document analysis to collect information on communication strategies used to promote childhood vaccination uptake in both states.

### Data collection: semi-structured interviews

Data collection took place from January to June 2014. We purposively selected stakeholders who had been involved in the development or delivery of communication strategies for vaccination at national, state, and local government levels as well as through developmental partners (e.g. UNICEF and WHO, Table 1).

**Table 1:** List of stakeholders interviewed

Level	Interviewees	Number of interviewees
National	Chief of Communication for Development, UNICEF	1
	National Immunization Officer for Communication, WHO	1
	GAVI representative	1
	Communication Analyst at the National Polio Emergency Centre	1
State	Social Mobilization Officer (State Health Educator): Two in Cross River and one in Bauchi	3
	Deputy Director, Community Health Services (Bauchi)	1
	State Immunization officer: Two in Cross River and one in Bauchi	3
	Deputy Director, Immunization Services (Bauchi)	1
	Volunteer Community Mobilizer (Bauchi)	1
	Traditional leader (Bauchi)	1
	Religious leader (Bauchi)	1
	‘Journalists against Polio’ Association representative (Bauchi)	1
Local	Local Immunization Officer (Bauchi or Cross River)	2
	Local Social Mobilization Officer (Bauchi or Cross River)	2
	Vaccinators (rural) (Bauchi or Cross River)	6
	Vaccinators (urban) (Bauchi or Cross River)	8
TOTAL		34

We used a semi-structured interview guide to gain insight into the vaccination communication interventions used for both routine vaccination and campaigns. The interview team was comprised of an interviewer and a note taker. Each interview lasted 30–45 min. We audio recorded each interview session after seeking and obtaining consent. At the end of each interview session, the recordings were transcribed verbatim and securely stored in a file bearing the date, place, and interview questions.

### Data collection: document review

We undertook a document review because interviewed participants may not have mentioned or remembered all the interventions being used, or may not have been aware of all of them. At the national and state levels, we collected relevant documents containing information on vaccine-related communication interventions. The documents included: policies on routine immunisation, routine immunisation strategic and implementation plans, reports on routine and supplemental immunisations, and ‘Reaching Every Ward’ plans.

### Data analysis

We commenced data analysis by going through each interview transcript and each document and extracting information about the communication interventions used for vaccination-related activities, the context in which they were used, the deliverer, and the purpose. After we had identified existing communication interventions, we used the COMMVAC taxonomy to categorise each intervention based on purpose (see below). We developed separate maps for Bauchi and Cross River states and then combined them to create a Nigerian map comprising all interventions in the two states organised by purpose and target audience. We presented the first draft of the map to some of the interviewees in both Cross River and Bauchi and asked them to add communication interventions that we had omitted and to clarify any unclear interventions. We also made an attempt to separate interventions used for routine immunisation and those used for campaigns.

### The COMMVAC taxonomy

The COMMVAC taxonomy was developed in response to the lack of a comprehensive approach to identifying and organising communication strategies or interventions used to improve childhood vaccination uptake (20) (Table 2). The taxonomy illustrates the relationships between different types of communication interventions and clarifies the key purposes and features of interventions to aid implementation and evaluation. The taxonomy was developed through a rigorous process of literature review and consultation with expert groups and draws on earlier taxonomies developed for communication interventions in general (11). The COMMVAC taxonomy includes seven main categories or purposes of communication interventions. These categories are broken down into several intervention types across three target groups: parents or soon-to-be-parents; communities, community members, or volunteers; and health workers.

**Table 2:** The ‘COMMVAC’ taxonomy – categories, definitions, and examples (20)

Taxonomy categories	Definition	Example
Inform or educate	Interventions to enable consumers to understand the meaning and relevance of vaccination to their health and the health of their family or community. Interventions are sometimes tailored to address low literacy levels and can also serve to address misinformation.	Educational sessions for parents and caregivers in their local health facility
Remind or recall	Interventions to remind consumers of required vaccinations and to recall those who are overdue.	Parent reminded through a mobile text message about their child's next vaccination appointment
Teaching skills	Interventions focussing on the acquisition of skills related to accessing and communicating about vaccination. Such interventions aim to teach parents early parenting skills such as how to find access and utilise vaccination services. They also include interventions to train parents, communities, and healthcare providers how to communicate or provide vaccination-related education to others.	Teaching people to critically appraise information and information sources through mothers’ groups
Provide support	Interventions, often tailored or personalised, to assist people in addressing specific challenges to vaccination that arise within their day-to-day lives (e.g. social issues such as disagreement within a family regarding vaccinating or emotional issues such as parental anxiety about vaccination.)	Biweekly parent support groups in the community or in health facilities
	In contrast to interventions to *inform or educate* about vaccination, interventions to provide support are more focussed on addressing specific challenges that people face when decided whether to vaccinate their child. However, interventions to provide support for vaccination may be combined with intervention to *inform or educate*.	
Facilitate decision-making	Interventions that extend beyond informing or educating by presenting all options related to vaccination decision-making in an unbiased and impartial manner. These interventions should provide detailed, evidence-based information about the risks and benefits of vaccination and should help people consider their personal values and options related to the decision to vaccinate their child.	Decision aid booklets sent to parents before a vaccination appointment
Enable communication	Interventions that explicitly and purposively aim to bridge a communication gap or make communication possible with particular people or groups. Generally, the translation of information into the languages of the primary target audience/s would not be included here as a specific intervention because this should be considered a basic implementation requirement. However, translation beyond routine practice in a particular setting such as adaptation of materials for a low- or no-literacy population, translation into braille, or the use of interpreters may be included.	Employment of translators in a clinic to facilitate communication
Enhance community ownership	Interventions to increase community participation and promote interaction between communities and health services. Interventions may build trust among consumers and generate awareness and understanding of vaccination. Interventions of this nature embrace collective decision-making and community involvement in planning, programme delivery, research, advocacy, or governance.	Organisations or community groups that consider the need for vaccines in their area discuss the costs and benefits of vaccination, and develop action plans to address barriers to uptake.

The development of the COMMVAC taxonomy was based largely on communication interventions from high-income settings, as this is where most of the published research to date on childhood vaccination communication has been conducted. The current study, as well as two other studies in Cameroon (27) and Mozambique, aimed to address this gap and improve the taxonomy.

### Ethical approval

We sought and obtained ethical approval from the ethical review committees in Cross River and Bauchi states.

## Results

A complete map of vaccination communication interventions for each state, organised by purpose, is available as a Supplementary table (Appendices 1 and 2). Table 3 provides examples of the interventions identified in each taxonomy category.

**Table 3:** Examples of communication interventions identified in Bauchi and Cross River states

Purpose of the communication intervention (organised using the COMMVAC taxonomy)	Examples of communication interventions employed in Bauchi State	Examples of communication interventions employed in Cross River State
Inform or educate	Health talks given by health workers before routine immunisation sessions in immunisation clinics	Educational sessions for parents and caregivers in their local health facility.
Remind or recall	Mothers and community members are reminded during home visits or house to house mobilisation by health workers of their next vaccination clinic appointment	Mothers and family members reminded of upcoming campaigns by health workers or through the use of town announcers
Teaching skills	Training of volunteer community mobilisers, traditional and religious leaders, to negotiate with non-compliant parents and provide adequate, correct and consistent information to community members	Frontline health workers or other immunisation providers are trained in interpersonal communication and negotiation skills to increase successful interactions with parents and caregivers
Provide support	No interventions identified	No interventions identified
Facilitate decision-making	No interventions identified	No interventions identified
Enable communication	No interventions identified	Health workers or community members who are employed as interpreters to help make communication possible in rural areas
Enhance community ownership	Women's groups, youth groups, and other community representatives involved in immunisation campaign days help teams identify missed children	Engagement of traditional or religious leaders and school teachers as advocates for vaccination

Most of the interventions we identified aimed to *inform and educate* people about childhood vaccination and to *remind and recall parents and communities about vaccination*. Fewer interventions aimed to *teach skills* and *enhance community ownership*. We did not identify any interventions in either state that aimed to *provide support* or *facilitate decision-making* or any interventions to *enable communication* in Bauchi State. Many of the interventions we identified had more than one purpose. The main targets of most interventions were caregivers and community members, with a limited range of interventions directed at health workers. In the sections below, we describe in more detail the interventions being used.

### Cross River State

#### Routine immunisation

*Interventions to inform and educate:* In Cross River State, most of the communication interventions we identified for routine immunisation aimed to *inform and educate* parents or soon-to-be-parents about childhood vaccination, and these interventions were usually delivered in the health facility. Commonly mentioned interventions to *inform and educate* included offering group health education to parents or pregnant women attending immunisation or antenatal clinics and the use of posters and flyers in clinics. In addition, health workers provided health education during home visits. These home visits were limited in terms of the number and range of interventions compared with home visits in the context of campaigns.

*Interventions to remind and recall:* Vaccination cards served as continuous reminders for vaccination appointments. Town announcers and radio announcements were also commonly used to remind communities about routine immunisation clinics in rural settings.

Parents of children whose immunisation schedules were not up to date were sometimes reminded of their vaccination appointments through phone calls and text messages from health workers. This was not routinely done as the cost was borne by the health workers.

*Interventions to teach skills:* During home visits, health workers taught mothers parenting and childcare skills. These included: how to care for vaccination sites, actions to take if vaccination side effects occurred, and the need for caregivers to ensure that each child completed her required vaccinations.

*Interventions to enable communication:* Health workers or community members who understood the native language were engaged as interpreters in primary health facilities in rural communities.

#### Immunisation campaigns

*Interventions to inform and educate:* By far the most common purpose of communication interventions used in immunisation campaigns in Cross River State was to *inform and educate*. The majority of these interventions were targeted at community members. Communication interventions aimed at informing and educating communities about campaigns were more common than routine immunisation activities and were delivered in a number of ways including: the use of town announcers, especially in rural settings; announcement letters sent to churches, mosques, traditional leaders, and schools; and the mass media, including jingles and announcements on radio and television. Other common interventions included community dialogues and community announcements by health workers. These interventions were frequently delivered just before immunisation campaigns. Printed materials such as posters and flyers were circulated in major health facilities in urban areas but delivery was inconsistent and funding dependent. Printed materials were rarely seen at the community level. Advocacy visits were frequently undertaken to relevant stakeholders in state and local governments, including relevant ministries or agencies (e.g. Ministries of Education, Women Affairs), just before a campaign to solicit their support and cooperation. Less frequently used interventions were market rallies and role plays in schools and communities.

Health workers occasionally received training, brochures, and fact sheets to update their knowledge base, but these interventions were dependent on the availability of funds.

*Interventions to remind or recall:* Community members were frequently reminded of upcoming campaigns through announcements in communities by health workers, town announcers, and the media (radio and television). Announcements were made in schools, churches, or mosques. Print materials such as posters, banners, and leaflets also served as reminders to parents and community members.

*Interventions to teach skills:* Vaccinators were sometimes trained in interpersonal communication and negotiation skills just before a campaign to equip them to communicate better with households and communities seen to be resistant to vaccination.

*Interventions to provide support:* We identified no interventions in this category.

*Interventions to facilitate decision-making:* We identified no interventions in this category.

*Interventions to enable communication:* Health workers who understood the native language or community-based volunteers were engaged as interpreters during vaccination campaigns in rural communities.

*Interventions to enhance community ownership:* We found few interventions which fell into this category. One example was ‘flag-off’ exercises by an influential political head for campaigns carried out at state and local government levels. Campaigns are preceded by advocacy visits to political and community opinion leaders to garner support. A ‘flag-off’ exercise marked the beginning of a campaign and was frequently undertaken in selected LGAs, especially those with low immunisation coverage rates. Also, the ward development committee and relevant community opinion leaders, such as market women leaders, youth leaders, traditional and religious leaders, the ward focal person, and local social mobilisation officers, came together to promote immunisation uptake.

### Bauchi State

#### Routine immunisation

The communication activities performed in Bauchi State in the context of routine immunisation were similar to those used in Cross River and so are not discussed again here. As in Cross River, these activities were fewer and less intensively implemented compared to similar activities in the context of campaigns.

#### Immunisation campaigns

*Interventions to inform and educate:* This was the most common category of interventions applied in Bauchi State for campaign purposes and targeted mainly community members. Commonly employed communication interventions included: engaging traditional and religious leaders; Quranic teachers; volunteer community mobilisers (lay health workers); polio survivors; organisations such as the Federation of Moslem Women Association of Nigeria, ‘Journalists against Polio’, and ‘Doctors against Polio’; as well as celebrity spokespeople (political or traditional leaders). These all served as polio vaccine advocates to sensitise community members. The mass media (e.g. community radio and television) was also frequently used to deliver information about immunisation campaigns. Bauchi State operates 10 community radio stations in the common local language (Hausa). A roadside film show conducted in communities in mobile vans (*majidi*) targeted beliefs about the cause of polio disease and negative attitudes towards polio. *Majidi* and community radio were commonly used in rural areas; and television messages were used in urban areas. Cassettes and CDs carrying vaccination messages, as well as print materials such as posters and banners, were also widely used. In addition, letters informing people about upcoming campaigns were sent to churches, schools, mosques, and traditional leaders; and announcements were made in churches, mosques, schools, and villages. Town hall meetings were common and targeted men in the community, whereas compound meetings (meetings involving several households) organised by Muslim women associations targeted women. Also, advocacy visits to relevant political and community opinion leaders were frequently conducted before campaigns to solicit support.

Less commonly used interventions included: drama troupes, media vans, market rallies, and community dialogues. Dialogues were used when there were issues of vaccine refusal or resistant households and aimed to inform the identified households of the importance of immunisation and why children should be brought for vaccination. These households were visited by a team including traditional or religious leaders, health workers, and a representative of ‘Journalists against Polio’ or polio survivors, depending on the reasons for refusal.

Training workshops were organised for health workers, and fact sheets and brochures were distributed to them just before a campaign.

*Interventions to remind or recall:* Caregivers and community members were reminded about upcoming immunisation campaigns through town announcers (particularly in rural settings) and other frontline communicators, including volunteer community mobilisers and health educators or social mobilisers. ‘Baby tracking’ by volunteer community mobilisers aimed to remind new mothers of the need to have their newborns vaccinated. Volunteer community mobilisers were required as part of their duties to monitor every new baby born in their settlement.

*Interventions to teach skills:* Community mobilisers, including health workers, were routinely trained to communicate and negotiate with vaccine-hesitant parents and communities and to provide adequate and consistent information to community members.

*Interventions to provide support:* We identified no interventions in this category.

*Interventions to facilitate decision-making:* We identified no interventions in this category.

*Interventions to enable communication*: We did not identify any example of interventions with the specific purpose of enabling communication. However, health talks, CDs, and print media used in rural settings conveyed vaccination messages in the local language.

*Enhance community ownership:* Efforts were made to enhance community ownership through ‘flag-off’ exercises in selected communities and through the use of local opinion leaders. Instances of partnership building and community coalition activities through ward health committees were also found. Youth and women leaders, and in some cases school teachers, accompanied vaccination teams and helped identify children who had not received polio vaccine in the last campaign.

## Discussion

A wide range of communication approaches for childhood vaccination have been adopted in Nigeria with the intention of improving vaccination coverage rates (7). The COMMVAC taxonomy allowed us to organise by purpose the complex range of interventions used in two states in Nigeria and was useful in identifying areas where communication efforts are concentrated and where gaps exist.

### Communication interventions for routine vaccination compared to vaccination campaigns

In both states, the use of communication interventions for routine immunisation was less frequent than the use of such interventions for campaigns. Interventions for routine immunisation were largely health facility based and delivered by health workers whereas a broader range of interventions were used for campaigns. This difference in the range and frequency of communication activities may be because of differences in donor or partner involvement. Such involvement in routine immunisation programmes is usually limited to technical components (e.g. logistics and management of cold chain facilities) (28), with limited funds allotted to communication efforts compared to campaigns for which external funding is frequently available. Communication strategies also have to compete for funding with other technical and operational aspects of the routine immunisation programme. This competition between communication strategies and other programme components was confirmed in the national strategic plan for routine immunisation as a reason for the programme's poor performance (18). Furthermore, the global drive to eradicate polio has made funds available from a larger number of non-governmental sources and has also tended to attract government resources away from routine immunisation activities (18), including communication activities.

### Range of vaccination communication interventions used

When the COMMVAC taxonomy was applied to the Nigerian context, it revealed that most communication interventions are clustered in two categories: *to inform and educate* caregivers and community members about immunisation and to *remind and recall*. This may be attributable to the fact that, in Bauchi, because of the lower immunisation coverage and resistance towards the polio vaccines, interventions targeted at informing and educating people of the benefits of vaccination and countering rumours and misconceptions surrounding polio were very common. Trusted community institutions were also used to deliver communication interventions and this may have enhanced community ownership. The taxonomy also identified gaps in communication activities. In both states, few communication interventions aimed to *teach skills* and *enhance community ownership* while no interventions aimed to *provide support* or *facilitate decision-making* in both states or *enable communication* in Bauchi. However, in Bauchi State, more communication approaches with a broader set of purposes were adopted and implemented, for the reasons described above.

### Target audiences for vaccination communication interventions

Another gap we identified was the lack of communication interventions directed at health workers. Health workers serve as an important source of information for the general public and are the main drivers of vaccination programmes (29, 30). However, most interventions directed at health workers were in the context of campaigns and few of such interventions appear to be used in the context of routine immunisation. As noted above, this may be related to the lower levels of funding available for these activities. It may also be tied to the fact that there is a general lack of attention to training health workers in interpersonal communication skills. This gap constitutes a missed opportunity to use encounters with health workers as communication events.

### Differences across and within Bauchi and Cross River states

Bauchi and Cross River states used similar communication activities for routine immunisation. For campaigns a wider range of interventions were employed in Bauchi, compared to Cross River, probably because of the increased need for and focus on immunisation coverage in the North. Most communication efforts in Bauchi aimed to *inform and educate* and *enhance community ownership* whereas, in Cross River, the interventions fell overwhelmingly into the *inform and educate* category. Moreover, in Bauchi most interventions employed were community-based and polio-driven, targeted households, and involved the use of appropriate channels to *inform and educate* mothers and community members. In addition to health workers, a range of other groups were engaged for the purpose of targeting caregivers at home in Bauchi, compared to Cross River. Targeting caregivers at home is important in Bauchi because in most Muslim communities women can only leave their home accompanied by their husbands. Fewer mobilisers were used to deliver communication interventions in Cross River, probably because this state has better vaccination coverage and because of the absence of polio. However, in both states the targets for these interventions were mainly community members.

Another difference observed was in interventions aimed at *enabling communication*, which were absent in Bauchi. This was because in Bauchi a common language was spoken by most people, whereas in Cross River, there is a wide diversity of languages and cultures.

Radio was used to deliver immunisation messages in both states but the intensity of its use was higher in Bauchi State. The media was strongly engaged to ensure that vaccination information had broad coverage, reaching hard-to-reach areas and migrant populations. Furthermore, radio is one of the most popular media in Nigeria and has widespread coverage across the country. It is often seen as an ideal medium for communicating with low-literacy communities, such as those found in certain areas of Northern Nigeria (31). ‘Work elsewhere’ has shown that such approaches may be useful (4), but further rigorous evaluations are needed (21).

The engagement of traditional and religious leaders as advocates for immunisation, especially targeting improving acceptance and uptake of polio in resistant communities, was used more visibly in Bauchi than in Cross River State. This may be because many cases of vaccination rejection in Bauchi, especially for polio vaccine, were for religious reasons and linked to rumours started by some religious leaders (32). Such intervention may be well accepted in this context and may contribute to reducing vaccine hesitancy (33). Similar interventions have also been adopted in other settings where vaccine hesitancy due to religious beliefs is common (34).

### Differences in communication interventions across rural and urban areas

Our study suggests that more interventions are targeted to rural communities, especially in polio-endemic areas. The use of community-based interventions was seen more frequently in high-risk rural areas of Bauchi State, compared to Cross River. In urban areas, the use of television was more prominent and printed materials were also more visible in urban health facilities.

### Using the COMMVAC taxonomy

We found the COMMVAC taxonomy to be a useful research tool, allowing us to create a sense of order across the complexity and range of communication strategies emerging from the fieldwork, and to examine which vaccination communication interventions are being used and where gaps in communication interventions exist. The taxonomy framework was also helpful when conducting interviews as it allowed us to present an organised map to participants, request feedback, and check the validity and completeness of the findings. The completed taxonomy also allows those working with vaccination communication to identify gaps in their own communication strategies because it can highlight relevant target audiences or purposes that they may have missed. By grouping the interventions by purpose in the map, programme managers can make sure that the interventions they use address key aspects of vaccine hesitancy in their local context, for example, those linked to lack of information or misinformation.

## Conclusions

The COMMVAC taxonomy was a useful research tool for analysing childhood vaccination communication interventions in the field. The tool allowed us to identify patterns in the communication interventions being implemented in two states of Nigeria at the time of the study. The tool also allowed us to uncover important gaps in relation to interventions used for routine immunisation, compared to campaigns. We found that most interventions aimed to *inform and educate* and *remind and recall* with limited interventions aiming to *teach skills*, *enhance community ownership*, or *enable communication*. We did not identify interventions to *provide support* or *facilitate decision-making*. Planners may wish to consider whether using interventions in these categories could contribute to addressing vaccine hesitancy in their setting. In addition, few interventions targeted healthcare workers. Training courses to update health workers’ interpersonal communication skills may enable them to communicate vaccination messages more effectively and should be considered by planners. The map of communication interventions also raises questions about caregivers’ views of communication interventions and how the interventions identified are being implemented, including barriers and facilitators to implementing vaccination communication interventions at scale. These questions will be addressed in forthcoming papers (27).

## Supplementary Material

Communication strategies to promote the uptake of childhood vaccination in Nigeria: a systematic map

## References

[1] CDC (1999). Ten great public health achievements – United States, 1900–1999.

[2] WHO (2014). Progress towards global immunization goals: summary presentation of key indicators.

[3] Bosch-Capblanch X, Banerjee K, Burton A (2012). Unvaccinated children in years of increasing coverage: how many and who are they? Evidence from 96 low-and middle-income countries. Trop Med Int Health.

[4] Obregón R, Ketan C, Morry C, Warren F, Jeffrey B, Galway M (2009). Achieving polio eradication: a review of health communication evidence and lessons learned in India and Pakistan. Bull World Health Organ.

[5] Mohammed AJ, Datta KK, Jamjoon G, Magoba-Nyanzi J, Hall R, Mohammed I (2009). Report on barriers to polio eradication in Nigeria.

[6] Andre F, Booy ER, Bock HL, Clemens J, Datta SK, John TJ (2008). Vaccination greatly reduces disease, disability, death and inequity worldwide. Bull World Health Organ.

[7] NDHS (2013). Nigeria demographic and health survey.

[8] Cobos Muñoz D, Monzón Llamas L, Bosch-Capblanch X (2015). Exposing concerns about vaccination in low- and middle-income countries: a systematic review. Int J Public Health.

[9] Waisbord S, Larson H (2005). Why invest in communication for immunization: evidence and lessons learned.

[10] Waisbord S, Shimp L (2010). Communication for polio eradication: improving the quality of communication programming through real-time monitoring and evaluation. J Health Commun.

[11] Hill S (2011). The knowledgeable patient: communication and participation in health.

[12] Oyo-Ita A, Nwachukwu C, Oringanje C, Meremikwu M (2011). Interventions for improving coverage of child immunization in low- and middle-income countries. Cochrane Database Syst Rev.

[13] Lewin S, Sophie H, Leyla HA, Sara BdCF, Xavier B-C, Claire G (2011). ‘Communicate to vaccinate’ (COMMVAC). Building evidence for improving communication about childhood vaccinations in low- and middle-income countries: protocol for a programme of research. Implement Sci.

[14] UNICEF (2012). UNICEF quarterly newsletter on polio eradication initiative in Nigeria. The Game Changer.

[15] NPHCDA (2008). National integrated communication and social mobilization strategy for immunization in Nigeria.

[16] GAVI (2014). GAVI alliance country tailored approach for Nigeria 2014–2018.

[17] Grade I (2013). Communication for Social Mobilization: an Evaluative Study of the National Immunization Campaign in Nigeria. J Human Soc Sci.

[18] FMOH, NPHCDA (2014). National routine immunization strategic plan 2013–2015 intensifying reaching every ward through accountability.

[19] Kaufman J, Synnot A, Hill S, Willis N, Horey D, Lin V (2013). Face to face interventions for informing or educating parents about early childhood vaccination. Cochrane Database Syst Rev.

[20] Willis N, Hill S, Kaufman J, Lewin S, Kis-Rigo J, De Castro Freire SB (2013). ‘Communicate to vaccinate’: the development of a taxonomy of communication interventions to improve routine childhood vaccination. BMC Int Health Hum Rights.

[21] Saeterdal I, Lewin S, Austvoll-Dahlgren A, Glenton C, Munabi-Babigumira S (2014). Interventions aimed at communities to inform and/or educate about early childhood vaccination. Cochrane Database Syst Rev.

[22] Ames H, Glenton C, Lewin S (2015). Parents’ and informal caregivers’ views and experiences of routine early childhood vaccination communication: qualitative evidence synthesis (Protocol). Cochrane Database Syst Rev.

[23] Kapp C (2004). Nigerian states again boycott polio vaccination drive. Muslim officials have rejected assurances that the polio vaccine is safe- leaving Africa on the brink of reinfection. Lancet.

[24] Shen A, Fields R, McQuestion M (2014). The future of routine immunization in the developing world: challenges and opportunities. Glob Health Sci Pract.

[25] Steinglass R (2013). Routine immunization: an essential but wobbly platform. Glob Health Sci Pract.

[26] WHO/USAID (2009). Periodic intensification of routine immunization: lessons learned and implications for action.

[27] Ames H, Njang D, Glenton C, Fretheim A, Kaufman K, Hill S (2015). Mapping how information about childhood vaccination is communicated in two regions of Cameroon: what is done and where are the gaps?. BMC Public Health.

[28] Uzochukwu B, Chukwuogo O, Onwujekwe O (2014). Financing immunization for results in Nigeria: who funds, who disburses, who utilizes, who accounts? Financing bottlenecks and accountability challenges. Afr J Health Econ.

[29] Eve D, Caroline L, Maryse G, Paul B, Réal R, Julie AB (2013). Vaccine hesitancy. Hum Vaccin Immunother.

[30] Schmitt HJ, Booy R, Aston R, Van Damme P, Schumacher RF, Campins M (2007). How to optimise the coverage rate of infant and adult immunisations in Europe. BMC Med.

[31] UNICEF (2012). Journalists initiative on immunization against polio in Nigeria.

[32] Nasir S-G, Aliyu G, Ya'u I, Gadanya M, Mohammad M, Mahmud Z (2014). From intense rejection to advocacy: how Muslim clerics were engaged in a polio eradication initiative in northern Nigeria. PloS Med.

[33] Nwaze E, Mohammed A (2013). An impact evaluation of the engagement of traditional and religious leaders in the Nigerian polio eradication initiative. Scholarly J Med.

[34] Jabbar A (2008). Religious leaders as partners in polio eradication [NWFP/FATA]. Technical Advisory Group on poliomyelitis eradication in Afghanistan and Pakistan.

