# Supplementary material for: Communication strategies to promote the uptake of childhood vaccination in Nigeria: a systematic map
Source: Glob Health Action. 2016 Feb 12;9:10.3402/gha.v9.30337. doi: 10.3402/gha.v9.30337 (PMC4754015; doi:10.3402/gha.v9.30337)
Supplement: Communication strategies to promote the uptake of childhood vaccination in Nigeria: a systematic map [file GHA-9-30337-s001.pdf]

Appendix 1: The 'Communicate to Vaccinate' taxonomy: Interventions to improve communication about childhood vaccination Bauchi

Red text = Interventions included in campaigns only
Blue text = Interventions included in routine vaccination only
Purple text = Interventions included in both campaigns and routine

		TARGET			
		**Parents or soon-to-be parents**	**Communities, community members or volunteers**		**Health professionals**
**PURPOSE**	**Inform or Educate**<br>Interventions to enable consumers to understand the meaning and relevance of vaccination to their health and the health of their family or community. Interventions are sometimes tailored to address low literacy levels and can also serve to address misinformation.	**One on one interactions**<br>Immunization information delivered by health workers on outreach services, home visits or clinic visits<br><br>**Group interactions**<br>Health talks given by nurses/ Community Health workers at immunization clinics in Health care facilities<br><br>Vaccination information given during Antenatal clinic visits to expectant mothers<br><br>Doctors against polio, Polio survivors, religious and traditional leaders provide vaccination information to parents.<br><br>**Device or tool**<br>Media van used for enlightenment.<br><br>**Audio visual / performance**<br>video clips, documentaries , CD plates given to parents on the importance of vaccination/immunization<br><br>-Use of Flip charts by VCMs in households.<br><br>**Printed material**<br>Use of Pamphlets, flyers, FAQs and fact sheets carrying vaccine/vaccination messages given to parents<br><br>-Announcements made in churches and mosques to encourage parents to present their wards for vaccination activities	**One on one interaction**<br>Volunteer community mobilizers (VCMs), health workers educate mothers in the community on the importance of having their wards vaccination.<br><br>**Group interactions**<br>Traditional and Religious leaders act as advocates to inform their communities on vaccination<br><br>Engagement of koranic teachers to disseminate key vaccination messages<br><br>Town hall meetings gather men in the community to sensitize them on the importance of vaccination.<br><br>Town announcers used to disseminate key vaccination messages in the community<br><br>Traditional leaders, Religious leaders , district heads, polio survivors, JAP, village health committee act as advocates to inform their communities about vaccination<br><br>-Federation of Muslim women Association (FOMWAN) holds compound meetings. Gather fellow women and talk to them about the importance of immunization<br><br>Advocacy visits to relevant stakeholders at state, Local and	**Audio visual / performance**<br>Community Radios and television announcements.<br><br>Majidi films at Community and viewing centres in Rural communities.<br><br>Use of cassettes and CDs carrying vaccination messages in local language distributed to community members<br><br>**Printed material**<br>Posters are printed and circulated in the community<br><br>Standard Announcement Letters sent to churches, mosques, district heads and schools to notifying them of upcoming campaigns<br><br>Billboards and banners strategically located and used during campaigns.<br><br>**Media campaign**<br>Vaccine info disseminated via radio or public address system/ mega phones on EPI vehicles.<br><br>Engagement of Drama troupes (edutainment) to demonstrate through drama the dangers associated with non-vaccination.<br>**Community event**	Fact sheets/ FAQs Brochures are produced to improve and update knowledge base of frontline health workers/vaccination teams<br><br>Training workshops, seminars and meetings targeting health professionals including health educators

			community levels<br>**Device or tool**<br>Face caps and T-shiirts<br>**Community Dialogues**<br>Community Meetings targeting heads of household (men)<br>Dialogues are held with Traditional, opinion and religious leaders in communities to address non-compliant cases and rumors.<br>**Mail (postcards, letters or email)**<br>Standard Announcement Letters sent to churches, mosques, district heads and schools to notifying them of upcoming campaigns<br><br>Use of traditional naming ceremonies / baby shows as opportunities to share key vaccination messages.<br>**Celebrity spokespeople**<br>e.g. Famous musician or Actor, political/ traditional or Religious leaders, Polio survivors serve as ambassadors to reinforce key messages on vaccination.	
	**Remind or Recall**<br>Interventions to remind consumers of required vaccinations and to recall those who are overdue.	**One on one interactions**<br>Mothers remind one another about immunization clinics and attend together<br>During home visits/house to house mobilization, Health workers remind mothers of their next RI clinic visit<br>**Phone-based (calls, hotlines or SMS)**<br>SMS and phone call reminders to Defaulting parents by health workers.<br>**device or tool**<br>Immunization card served as a cue to remind parents of next vaccination	Use of mega phones by health worker, media vans, media to remind members of the community on upcoming campaign or routine immunization dates.<br>Key Frontline Communicators,(VCMs, JAP, health educators, TBAs. Polio survivors) community based organization (CBOS) such as Federation of Muslim women association and encourage and remind parents in the community on need to immunize children.<br>Town announcers remind members of the community of upcoming campaigns or immunization clinics days in certain LGAs<br>Baby tracking and monitoring of newborns by voluntary community mobilizers during traditional naming ceremonies serves as a reminder to parents<br>Announcement made in churches/ mosques/schools<br>TV/ radio announcements<br>Banners, posters and flyers used during campaigns	**One on one interaction**.<br>Community health workers checks for immunization status at every visit and verbally prompts health worker in charge of the facility.

PURPOSE				
	**Teach Skills**† Interventions focusing on the acquisition of skills related to accessing and communicating about vaccination. Such interventions aim to teach parents early parenting skills such as how to find access and utilise vaccination services. They also include interventions to train parents, communities and health care providers how to communicate or provide vaccination-related education to others.	**Training on how to communicate/ provide education to others**<br>Mothers (women groups, VCMs, religious leaders) are identified and trained to provide information to parents especially in resistant and high risk communities<br>**Parenting skills programs**	**Training in how to communicate/ provide education to others**<br>Training of Volunteer community mobilizers, traditional, religious leaders, and traditional birth attendants how to better communicate, negotiate with noncompliant parents and provide adequate, correct and consistent information to community members.<br>Refresher courses organized for journalists. E.g. journalist against polio network (JAP) to update their knowledge base on vaccines/vaccination issues.	**Training on how to communicate/ provide education to others**<br>Front line Health workers /other immunization front liners are trained on Interpersonal communication and negotiation skills to increase successful interactions
	**Provide Support**† Interventions, often tailored or personalised, to assist people in addressing specific challenges to vaccination that arise within their day-to-day lives (eg social issues such as disagreement within a family regarding vaccinating or emotional issues such as parental anxiety about vaccination.)<br>In contrast to interventions to inform or educate, interventions to provide support are more focused on addressing specific challenges faced by parents when making vaccination decisions.			
	**Facilitate Decision Making** Interventions that extend beyond informing or educating by presenting all options related to vaccination decision-making in an unbiased and impartial manner. These interventions should provide detailed, evidence-based information about the risks and benefits of vaccination and should help people consider their personal values and options related to the decision to vaccinate their child.	**Decision aids**		**Decision aids**
	**Enable Communication** Interventions that explicitly and purposively aim to bridge a communication gap / make communication	**Interpreters**		

	possible with particular people or groups. Generally, the translation of information into the languages of the primary target audience/s would not be included here as a specific intervention because this should be considered a basic implementation requirement.				
	**Enhance Community Ownership**<br>Interventions to increase community participation and promote interaction between the community and health services. Interventions may build trust among consumers and generate awareness and understanding of vaccination. Interventions of this nature embrace collective decision making and community involvement in planning, program delivery, research, advocacy or governance.	**Program delivery**<br>Flag off exercises conducted in LGAs to officially start off an immunization campaign	**Local opinion leaders**<br>Vaccine information delivered by village heads or religious leaders<br><br>Engagement of traditional, religious, community members, mobilizers, Quranic teachers as immunization advocates in their communities.<br><br>Volunteer community mobilizers(VCMs) educate mothers in the community on the importance of vaccination<br><br>**Community input**<br><br>Community dialogues to address non-compliance, AEFI and rumors<br><br>Advocacy visits to community opinion formers(religious, traditional, youth and women groups)	**Program delivery**<br>Local NGOs involved in EPI or immunization campaign days<br><br>**Community coalition**<br>Community coalition including community members and health providers work with state and local government to implement vaccine programmes<br><br>**Partnership building**<br>Community partnerships among existing structures (CBOs, trade associations, religious groups, traditional rulers, extended families to act as change agents as well as beneficiaries.	

Appendix 2:

The 'Communicate to Vaccinate' taxonomy: interventions to improve communication about childhood vaccination: Cross river state

Red text = Interventions included in campaigns only
Blue text = Interventions included in routine vaccination only
Purple text = Interventions included in both campaigns and routine

		TARGET			
		Parents or soon-to-be parents	Communities, community members or volunteers		Health professionals
PURPOSE	**Inform or Educate**<br>Interventions to enable consumers to understand the meaning and relevance of vaccination to their health and the health of their family or community. Interventions are sometimes tailored to address low literacy levels and can also serve to address misinformation.	**One on one interactions**<br>Immunization information delivered by health workers on outreach services, home visits or clinic visits<br><br>**Group interactions**<br>Health talks given by nurses/ Community Health workers at immunization clinics in Health care facilities<br><br>Vaccination information given during Antenatal clinic visits<br><br>**Device or tool**<br><br>**Audio visual / performance**<br>Jingles, Press releases and Public service announcement and Documentaries to parents on the importance of vaccination/immunization<br><br>**Printed material**<br>Use of Pamphlets, flyers, FAQ and fact sheets carrying vaccine/vaccination messages given to parents<br><br>Posters, leaflets and flyers are also used<br><br>Billboards and banners strategically located to inform people about upcoming campaign.<br><br>-Announcements made in churches and	**One on one interactions**<br>Home visits by health workers to mothers in the community.<br><br>**Group interactions**<br>Advocacy visits to relevant stakeholders at state, Local and community levels including relevant ministries<br><br>Town announcers used to disseminate key vaccination messages in the community<br><br>Youth/ Women's group meetings.<br><br>Focus group discussions held in women's meetings within the community<br><br>-Role play carried out in schools to inform children<br><br>Health worker makes announcements to community members using megaphones<br><br>**Community Dialogues**<br><br>Dialogues are held with Traditional, opinion and religious leaders in communities to address non-compliant cases and rumours, AEFI.<br><br>**Mail (postcards, letters or email)**<br>Standard Announcement Letters sent to churches, mosques and schools to promoting vaccination	**Audio visual / performance**<br>Radio and television announcements, documentaries, press releases, phone in programs<br><br>**Printed material**.<br>Posters printed and circulated in the community by NGOs<br><br>**Media campaign**<br>Vaccine info disseminated via mega phones on EPI vehicles.<br><br>**Community event**<br>Market rallies conducted in specific LGAs on market days.<br><br>**Celebrityspokespeople**<br>.	Fact sheets/ FAQs Brochures are produced to improve and update knowledge base of frontline health workers/vaccination teams<br><br>Training workshops, seminars and meetings target health professionals including health educators

		mosques to encourage parents to present their wards for vaccination activities	**Market Rallies:** held to sensitize members of the community on an upcoming mass campaign	
	**Remind or Recall**<br>Interventions to remind consumers of required vaccinations and to recall those who are overdue.	**One on one interactions**<br>Mothers remind one another about immunization clinics and attend together<br>During home visits/house to house mobilization by Health workers mothers are reminded of their next RI clinic visits<br>**Phone-based (calls, hotlines or SMS)**<br>SMS and phone call reminders to parents, Defaulting parents by health workers.<br>Town announcers remind parents of upcoming campaigns or immunization clinics days in certain LGAs<br>**Device or tool**<br>Immunization card serves as a cue to remind parents of next vaccination	Use of mega phones by health worker to remind member of the community on upcoming campaign or routine immunization dates.<br>Key Frontline Communicators, community based organization (CBOS) ,health educators and TBA encourage and remind parents in the community on need to immunize children.<br>Town announcers remind members of the community of upcoming campaigns or immunization clinics days in certain LGAs Announcement made in churches/ mosques/schools<br>TV/ radio announcements<br>Banners, posters and flyers used during campaigns	**One on one interactions**<br>Nurses checks for immunization status at every visit and verbally prompts doctor or health worker in charge of the facility.
**PURPOSE**	**Teach Skills**<br>Interventions to provide individuals with the ability to operationalise knowledge through the adoption of practicable skills.	**Training on how to communicate/ provide education to others**<br>**Parenting skills programs**<br>Home visits to teach parenting skills and child health	**Training in how to communicate/ provide education to others**<br>The traditional and religious leaders, women representative, Town announcers are trained on how to deliver key vaccination messages to others.	**Training on how to communicate/ provide education to others**<br>Front line Health workers /other immunization front liners are trained on Interpersonal communication and negotiation skills to increase successful interactions
	**Provide Support**<br>Interventions to provide assistance or advice for consumers outside the traditional consultation environment.			

	**Facilitate Decision Making**<br>Interventions that extend beyond informing or educating by presenting all options related to vaccination decision-making in an unbiased and impartial manner. These interventions should provide detailed, evidence-based information about the risks and benefits of vaccination and should help people consider their personal values and options related to the decision to vaccinate their child	**Decision aids**		**Decision aids**
	**Enable Communication**<br>Interventions that explicitly and purposively aim to bridge a communication gap / make communication possible with particular people or groups. Generally, the translation of information into the languages of the primary target audience/s would not be included here as a specific intervention because this should be considered a basic implementation requirement.	**Interpreters**<br>Employment of Health workers/ community volunteers(indigenes of the community) to act as interpreters during health education sessions in certain rural communities		
	**Enhance Community Ownership**<br>Interventions to increase community participation and promote interaction between the community and health services. Interventions may build trust among consumers and generate awareness and understanding of vaccination. Interventions of this nature embrace collective decision making and community involvement in planning, program delivery, research, advocacy or governance.	**Program delivery**<br>Flag off exercises conducted in specific LGAs to officially start off an immunization campaign	**Local opinion leaders**<br>Engagement of traditional, religious, farmers, teachers, women /youth leaders as immunization advocates in their communities.<br>**Community input**<br>Focus groups to seek recommendations from the community about how to better serve the community<br>Community dialogues to address non-compliance, AEFI and rumors<br>Advocacy visits to community opinion formers(religious, traditional, youth and women groups<br>**Program delivery**<br>Local NGOs involved in EPI or immunization campaign days<br>**Community coalition**<br>Community coalition including community members and health providers work with state and local government to implement vaccine programmes<br>**Partnership building**<br>Community partnerships among existing structures (CBOs, trade associations, religious groups, traditional rulers, extended families to act as change agents as well as beneficiaries.	
